# Hereditary Insanity

**Published:** 1882-08

**Authors:** A. W. Hagenbach

**Affiliations:** Assistant Superintendent Cook County Hospital for the Insane


CHICAGO MEDICAL

JOURNAL & EXAMINER.

VOL. XLV.—AUGUST, 1882.—NO. 2.

### Original Communications.

ARTICLE I.

HEREDITARY INSANITY. By A. W. HAGENBACH, M.D., Assistant Superintendent Cook County Hospital for the Insane.

The large percentage of insane patients admitted to this hospital in whom there pre-existed an undoubted hereditary predisposition to insanity led me, several years ago, to begin paying special attention to that part of the histories relating to heredity. As I have been stationed in the male department, the following observations will relate more especially to the male insane. A few cases among females will, however, be made use of to illustrate certain points.

I have before me the histories of 150 male patients admitted to the hospital from September 1, 1879, to September 1, 1881, and from these most of my deductions are drawn. My reasons for limiting myself to these cases are the brief and unsatisfactory histories of patients previously admitted; also because I preferred accurate histories in limited numbers, to the promiscuous statistics too frequently made use of by the enthusiastic statistician. The difficulties encountered in obtaining accurate family histories cannot be appreciated by anyone who has not had occasion to collect them. People will frequently insist that every one of their blood relatives enjoyed excellent general health, and a most desirable mental and nervous constitution, while their speech and actions completely refute their assertions. The mere statement by relatives, that "There is no insanity in our family," is far from conclusive, as not infrequently, when more specific inquiries are made into the family history, it will be learned that an uncle, or aunt, committed suicide without any known cause, while a cousin was always regarded as "peculiar," and still others were epileptic, hysterical, or what not, and yet they cannot imagine how a member of such a healthy and well balanced family could ever have become insane. Ignorance of family history, false pride, and even superstitious fears have at times to be contended with in collecting histories.

The following table, "Table I," will show the age, occupation, form of insanity, and number and degree of relationship of insane relatives, of all the insane, in whom a history of hereditary predisposition was obtained, in the one hundred and fifty patients of whom this article chiefly treats.

In glancing over the table, it will be seen that 41 patients out of 150, admitted during two years, had insane relatives. If we subtract from this number the five cases who had insane cousins only as insufficient evidence of heredity, as the insanity might have been transmitted to them by persons related to the patient by marriage only, we still have 36 patients with a strong history of an inherited predisposition to insanity. When it is remembered that these patients were inmates of a pauper asylum, that at times it is impossible to learn even their correct names, and the relatives when found were frequently lamentably ignorant of their family histories, there can be no doubt that could the full histories of all the patients have been ascertained, fully 33 per cent. would have had insane relatives, and are themselves the victims of an inherited predisposition to mental disease.

TABLE I.

| No. | Age. | Occupation. | Form of Insanity. | Relatives Insane. | | | | | | | | | |
|---|---|---|---|---|---|---|---|---|---|---|---|---|---|
| | | | | Father. | Mother. | Sister. | Brother. | Uncle. | Aunt. | G. Mother. | G. Aunt. | Cousin. | |
| 1 | 45 | Engineer. | Melanch. | 1 | .... | 4 | .... | 1 | 1 | .... | .... | .... | |
| 2 | 18 | Laborer. | Mania. | .... | .... | .... | .... | .... | 1 | .... | .... | .... | Both parents very intemperate. |
| 3 | 34 | Exp'sman. | G. Paresis. | .... | .... | .... | 1 | .... | .... | .... | .... | .... | |
| 4 | 24 | Clerk. | Dementia. | .... | .... | .... | .... | 1 | .... | .... | 1 | 1 | Mother extremely nervous. |
| 5 | 48 | Jockey. | Melanch. | .... | 1 | .... | 1 | .... | .... | .... | .... | .... | A son epileptic. |
| 6 | 52 | Laborer. | Dementia. | 1 | .... | .... | .... | .... | .... | .... | .... | .... | |
| 7 | 33 | Peddler. | Melanch. | .... | .... | .... | .... | 3 | 1 | .... | .... | .... | Mother paralyzed. |
| 8 | 39 | B'ksmith. | Melanch. | .... | .... | 1 | 1 | .... | .... | .... | .... | ... | |
| 9 | 29 | Laborer. | Mania. | .... | .... | .... | 1 | .... | .... | .... | .... | .... | Two nephews imbecile. |
| 10 | 34 | Laborer. | Melanch. | .... | 1 | .... | .... | .... | .... | .... | .... | .... | |
| 11 | 32 | Printer. | Mania. | 1 | .... | .... | .... | .... | 1 | .... | .... | .... | Parents were first cousins. |
| 12 | 39 | L'ksmith. | Dementia. | 1 | .... | .... | .... | .... | .... | .... | .... | .... | |
| 13 | 50 | Laborer. | Man. Rec. | 1 | .... | .... | .... | .... | .... | .... | .... | .... | |
| 14 | 22 | Cigarmkr. | Dementia. | .... | .... | .... | .... | .... | .... | .... | .... | 1 | |
| 15 | 54 | Hospter. | Neu Epil. | .... | .... | .... | .... | .... | .... | .... | .... | 1 | |
| 16 | 28 | Uph'lste'r. | Melanch. | .... | .... | .... | 1 | .... | .... | .... | .... | .... | |
| 17 | 23 | Liverym'n | Melanch. | .... | .... | .... | .... | .... | .... | .... | ... | 1 | |
| 18 | 26 | Student. | Melanch. | 1 | 1 | .... | 1 | .... | .... | 1 | .... | .... | Brother and grandmother suicidal. |
| 19 | 52 | Plasterer. | Mania. | 1 | .... | .... | .... | .... | .... | .... | .... | .... | |
| 20 | 29 | Printer. | Man. Rec. | 1 | ... | .... | .... | .... | 1 | .... | .... | .... | |
| 21 | 40 | Laborer. | Mania. | .... | .... | .... | .... | .... | 1 | .... | .... | 1 | |
| 22 | 32 | Laborer. | Dementia. | .... | .... | .... | .... | .... | 1 | .... | .... | 1 | Two nephews insane. |
| 23 | 30 | Shoem'k'r | Melanch. | .... | .... | .... | 1 | .... | .... | .... | .... | .... | |
| 24 | 35 | Clerk. | Mania. | .... | .... | 1 | .... | .... | .... | .... | .... | ... | Father died of cerebral apoplexy. |
| 25 | 30 | Clerk. | G. Paresis. | .... | .... | .... | .... | 1 | .... | .... | .... | .... | |
| 26 | 66 | Farmer. | Melanch. | .... | 1 | .... | .... | .... | .... | .... | .... | .... | Father died of paralysis agitans. |
| 27 | 24 | Actor. | Melanch. | .... | .... | .... | .... | 1 | .... | .... | .... | .... | |
| 28 | 32 | Laborer. | Dementia. | .... | .... | .... | .... | .... | 1 | .... | .... | .... | |
| 29 | 62 | Machinist. | Melanch. | .... | .... | .... | 11 | .... | .... | .... | .... | .... | Two brothers committed suicide. |
| 30 | 33 | Laborer. | Man. Rec. | .... | .... | .... | 1 | .... | .... | .... | .... | .... | |
| 31 | 50 | Laborer. | Dementia. | .... | .... | .... | .... | 1 | 1 | .... | .... | .... | |
| 32 | 40 | Farmer. | Melanch. | .... | .... | .... | .... | .... | .... | .... | .... | 1 | Father very eccentric. |
| 33 | 38 | Laborer. | Man. Rec. | .... | .... | 1 | 1 | .... | 1 | .... | .... | .... | |
| 34 | 35 | Painter. | Mania. | 1 | .... | .... | .... | .... | .... | .... | .... | .... | |
| 35 | 36 | Baker. | Mania. | .... | .... | .... | .... | 1 | .... | .... | .... | 1 | |
| 36 | 45 | Printer. | Dementia. | .... | .... | .... | .... | .... | .... | .... | ... | 1 | |
| 37 | 39 | Clerk. | G. Paresis. | .... | 1 | .... | ... | .... | .... | .... | .... | 11 | Father hemiphlegic. |
| 38 | 27 | Laborer. | Dementia. | .... | 1 | .... | .... | .... | .... | .... | .... | .... | |
| 39 | 41 | Fireman. | Mania. | .... | .... | 1 | .... | .... | .... | .... | .... | ... | |
| 40 | 45 | Carpent'r. | Man. Rec. | .... | .... | .... | .... | 1 | .... | .... | .... | 1 | |
| 41 | 37 | Clerk. | Melanch. | .... | .... | .... | 1 | .... | .... | .... | .... | .... | |
| | | | Total..... | 9 | 6 | 8 | 12 | 10 | 10 | 1 | 1 | 12 | |

The fact that different writers on insanity report from 15 to 75 per cent. of the admissions to asylums as due to an inherited predisposition, may possibly be accounted for by the various area of relationship included in these tables, as well as the more or less complete facilities for getting histories of the cases admitted. It would not be remarkable that the percentage regarded as hereditary should be larger in private than public asylums, because the relatives of the class of patients usually sent to private hospitals are better informed as to their family histories than those of the average indigent lunatic.

In reply to the question, "How does the insane neurosis originate?" I have become satisfied that some of the chief elements are intemperance in ancestors, consanguineous marriages, intermarriages of persons of like vicious temperaments, and tuberculosis. I could enumerate a number of cases of intemperance in ancestors resulting in insanity in the offspring, as this is a frequent item in the histories of the insane, but one illustrative case will be briefly reported.

Case 1.—J. C., after some slight exciting cause, went violently insane. When admitted to the hospital all the symptoms of acute mania were well marked. In taking a history of the case it was learned that his ancestors on paternal and maternal sides were long lived, and well balanced; there had never been a single case of insanity or other nervous affection in either branch, so far as could be ascertained. As he had always enjoyed average health previous to the attack of insanity, which had manifested itself after a slight quarrel with his aunt, I inquired very carefully into the family history, but was unable to learn any facts tending to show that his ancestors could have been responsible for his poorly balanced mind, until I learned from outside sources a fact which had been carefully concealed by his relatives, viz., his father was a habitual drunkard for years previous to the birth of the patient, and died of delirium tremens. His mother, who was still living at the time of his admission, had also indulged to excess before the patient was born. He commenced to improve shortly after his admission, and was discharged as "recovered" in a few days less than eight months, and has been working ever since. The mental excitement for the time was allayed, but unless he is extremely fortunate in escaping all sources of excitement, the probabilities are that he will sooner or later again become deranged, and most likely end his days as a chronic dement in some hospital for the insane, as each recurrence of the maniacal excitement will leave his mind more and more shattered, and less able to resist exciting causes, or recover from the maniacal excitement.

*Consanguineous Marriages.*—"Like begets like." The parents are reproduced in the children, transmitting to them all their qualities, whether good or bad. If both parents have similar bad qualities, the offspring inherit their combined defects. This fact is understood by the intelligent breeder, who carefully studies all the good and bad points of the animals he is about to breed. But a short time ago a gentleman refused to breed a cross mare with an equally vicious horse, because he feared the offspring would be so refractory as to be entirely unmanagable. Different breeds of horses have distinguishing traits by which they are easily recognized. I remember a steed whose colts, nearly without exception, it was almost impossible to break to drive, but if once thoroughly broken they made the safest of horses. Different breeds of horses have characteristic mental traits, such as gentleness, kindness, etc., by which they may be recognized almost as surely as by their peculiarities of form, color or gait. While interbreeding within narrow limits is sometimes resorted to, to increase certain desirable points, if carried too far the breed invariably deteriorate. In the human species, the same as among the lower animals, interbreeding in certain cases might prove harmless or even beneficial to the offspring, but such cases must necessarily be as rare as the examples of families who are entirely free from all nervous and physical de-defects.

To illustrate how consanguineous marriages may prove harmless in one instance, and deleterious in another, I report the two following cases, which have come under my own observation. With all the parties of the first case I have been intimately acquainted for years. The second case came to my notice in this institution several years ago.

Case II.—A, aged about forty years, of sanguine temperament, married his first cousin, B, aged about twenty-five years. As a result of the marriage four children were born, one boy and three girls, all of whom are living and enjoying good physical and mental health. The father of A, of a nervo-sanguine temperament, a school teacher by profession, died at the age of eighty-nine years, of "general exhaustion accompanying old age." His mental faculties at the age of eighty-six, when I last saw him, were remarkably well preserved. The mother of A, of a mixed temperament, always enjoyed good health, and at the age of eighty-five years was still quite hale and hearty, and in full possession of all her mental faculties. Her memory was remarkably retentive for one of her years, and she could repeat from memory the names and dates of all her grandchildren, over twenty in number. Her recollection of recent events was quite remarkable. After reading a newspaper she could repeat correctly all the items of interest, not omitting the most minute particulars, even to the dates, localities, etc. She died of "general exhaustion accompanying old age," aged eighty-eight years.

The father of B, a sober, industrious man, of a phlegmatic temperament, died, aged sixty-five years. Cause of death unknown. The mother of B, a sister of A's mother, died at the age of seventy-five years, of "diarrhœa and old age." The family histories of all concerned were remarkably free from nervous affections, not a single member, so far as could be ascertained, had ever been insane, epileptic or hysterical, neither had there been a single death from rheumatism, phthisis pulmonalis, or cancer, in the entire family. It will be seen by reviewing the histories of A and B, that they should have inherited good mental and physical health, and their children, perfect in every respect, demonstrate that the danger of consanguineous marriages to the offspring does not depend upon the degree of consanguinity, but upon the increase of defects (especially nervous) existing in the parents.

Case III.— C, aged twenty-five, of nervous temperament, whose father was very nervous and eccentric, married his first cousin, also of a nervous temperament. The mother of D, and sister to C's father, although never confined in an asylum, was regarded by every one as of unsound mind for many years previous to her death. Result of marriage, five children, two girls and three boys. The boys all died during the period of dentition. Two died in convulsions. Of the two girls, one died in the hospital, of epileptic dementia, the other, and only surviving child, aged about eleven years, was a microcephalic idiot, who had never been able to walk, on account of deformed extremities, and when I last saw her parents, about three years ago, I was informed that she was entirely helpless, unable to feed herself, or even direct attention toward the calls of nature.

To the question so frequently asked of the physician, "Are consanguineous marriages harmful?" I would reply, in the words of Prof. J. Adams Allen, "That depends upon the mental and physical condition of the parties united in marriage." There are but very few families that are entirely free from all nervous defects. This I consider as one of the greatest reasons why consanguineous marriages generally prove harmful, and the miserable creatures frequently begotten are such burdens to themselves and society, and the danger of latent nervous disease even in persons of apparently good health are so great, that I would favor the passage of laws strictly prohibiting all consanguineous marriages. The cases of the patriarchs who intermarried very closely cannot, I think, be cited as favoring the practice, as from all accounts extant of these people, we are led to regard them as persons who arrived at very great ages, enjoyed excellent general health, and were remarkably clear-headed. In short, they were just the people who could stand intermarriage with blood relatives. But the ordinary mortal of the nineteenth century, afflicted with inherited neuroses, should not, in this respect, take pattern by the examples set by the patriarchs. There are few more difficult problems for the physician to solve than that of the absence or presence of danger to the offspring in relatives about to be united, and unless satisfied by a careful examination of the parties, as well as the family histories, that there is no possible danger the union should be strongly objected to.

*Other Improper Marriages.*—If not somewhat foreign to my subject, a great deal might be said regarding the duties of physicians when called upon to express a professional opinion, regarding the advisability of parties becoming united by marriage, not related, but affording evidences of insanity, phthisis pulmonalis or any other defect that could endanger the health or existence of the offspring. Physicians too frequently pander to the popular delusions that puberty and marriage are the panaceas for every ailment to which the young female is subject to. The parents of nervous or scrofulous children are promised a restoration to health after the establishment of the menstrual function, and if no improvement follows at this period, the next step is to select some rugged mate, who is to effect some wonderful transformation in the unfortunate being. A sad case of this kind came to my notice in the person of F. B., an inmate of this asylum, who was imbecile and hysterical since ten years of age. At the age of seventeen years she was given in marriage by her parents to a drunken, worthless fellow, who, after living with her for several years, ill-treating her in a shocking manner, deserted her, a mother of two children, which, fortunately for themselves and society, died in infancy and early childhood. By removing from society these imperfect creations, preventing the propagation of so deleterious an element, and affording protection to those who, above all others, are easily misled and imposed upon, the hospitals for Feeble-Minded Children, which have been established in the various States of the union, perform their highest mission.

Phthisis pulmonalis is a very common cause of death among the ancestors of the insane. In fifty cases of insanity where the causes of death of parents were pretty accurately ascertained eight had fathers and seven had mothers die of phthisis pulmonalis. A patient, at present an inmate of this hospital, with evidences of tubercular deposits in both lungs, and whose insanity is regarded as due to tubercular meningitis, had his father, brother, four first cousins and paternal uncle all die of phthisis pulmonalis. Phthisis is one of the most frequent causes of death among the insane, and as the usual signs, such as cough and expectoration, are frequently greatly modified or entirely wanting, patients are not infrequently found, upon physical examination, in an advanced stage of the disease, in whom its presence had hardly been suspected. In a large percentage of post-mortems held upon the chronic insane, tubercular deposits were found (generally in the lungs only), but in several instances greatly diffused throughout the entire body, especially in the peritoneum, and cerebral meninges.

Aside from the causes already enumerated, any disease in the parents tending to innervation and general debility, may have an effect upon the nervous organization of the children begotten while the parents are thus debilitated.

According to some of the best authorities, insanity in the mother is more dangerous to the daughters than the sons, while insanity in the father is said to be about equally dangerous to sons and daughters, and that on the whole insanity in the mother is more dangerous to the offspring than insanity in the father.

By glancing at Table I. it will be seen that nine of the patients had insane fathers, and six had insane mothers. That insanity in the mother is more dangerous to the offspring can, I think, be explained, in part, at least, by remembering the relative importance of the two sexes in the reproduction of the species, especially the human, in whom the fœtus, for months, is carried in the uterus of the female, and, I firmly believe, influenced to a certain extent by the emotions and nervous impressions of the mother; and for months after birth the child receives its sole nourishment from the female lacteal secretions, which certainly are changed in quality and quantity by the mental condition of the mother, and even later, during the early years of childhood, while the characters are being formed, the mother, by her constant presence and example, almost invariably exerts the greater influence over the offspring. Of the five great acts comprising the function of generation, 1, copulation, 2, fecundation, 3, gestation, 4, delivery, 5, lactation, the male takes part in the first two only. While the relative importance of the sexes may be reversed in certain of the batrachia, where impregnation takes place after the spawn in which the eggs are contained are deposited outside of the body of the female, in the mammalia the female performs the greater part of the function of generation.

There was a time when breeders imagined that all it was necessary to do to improve their stock was to import a blooded bull, but a radical change of opinion has taken place among intelligent breeders, as can be plainly seen by visiting a blooded cattle show and noticing the great importance attached to the pedigree of heifers and cows, also the increasing demand and high prices of thoroughbred heifers and cows.

Is the form of insanity or an insane neurosis transmitted? I have seen no cases in which I believed the form of insanity had been transmitted, but regarded all the cases that came under my care as having inherited an unstable nervous system, with a predisposition to nervous, and especially mental disease, the form of insanity depending chiefly upon surroundings, education, and exciting causes. Thus, of two brothers inheriting an insane temperament, one by excessive mental work and close confinement, impairs his general health, becomes dyspeptic, anæmic and neuresthenic. If, while in this condition he becomes insane, the insanity is apt to be less acute and less amenable to treatment than insanity in the brother, who always enjoyed active out-door exercise and good digestion until he met with some sudden reverse in fortune, which disturbed the equilibrium of his inherited poorly balanced mind.

The cases of suicidal melancholia reported, in which several members of the same family became melancholy and committed suicide, I do not regard as an evidence of the theory of the transmission of the form of insanity, and think they can be explained by remembering that "The particular form of insanity is determined by the natural temperament, and as the temperament is hereditary, the predisposition to insanity in blood relatives being similar in nature, will result in similar forms of insanity," modified by surroundings, education, etc.

One of the most remarkable histories of an inherited predisposition to insanity ever obtained in a patient admitted to this hospital, is case 1, of Table I, and is briefly reported as affording an example of the virulent nature of the neurosis in some families.

Case IV.—H. F., aged forty-five years, locomotive engineer, admitted September 11, 1879. Upon examination found him poorly nourished; heart and lung sounds normal; tongue pale and flabby. Partial paralysis involving right side, of several weeks' duration. For cranial measurement, etc., see case 1, Tables I and III. Mentally he was alternately melancholy and maniacal, with short intervals of comparative sanity from the time of his admission until he eloped, January 15, 1880. He was the youngest of seven children, four of whom died insane. The following enumeration will show the present condition of those living, and causes of death of those deceased, of all his brothers and sisters:

I.—C. F., farmer, living, mental condition unknown, general health has been greatly impaired for many years.

II.—B. F., died, aged nineteen years, from sequelæ of measles.

III.—W. F., lake captain, aged fifty-five years, suffers greatly with nervous headache, neuralgia, and marked impairment of his general health. Very nervous and emotional.

IV.—Sister, age unknown, for many years an inmate of the State Hospital for Insane, at Utica, N. Y. Was removed from the hospital completely demented several weeks previous to her death. Form of insanity, melancholia.

V.—Sister. Died in asylum at Rochester, N. Y.; age, form of insanity and cause of death, unknown.

VI.—Sister; married; died at her home of puerperal insanity several weeks after confinement.

VII.—C. F., subject of this history.

His father, although never an inmate of an asylum, was of unsound mind for ten years previous to his death. His paternal uncle was an inmate of the asylum at Utica, N. Y., for several months, and was discharged as recovered in 1853. He afterward held numerous positions of trust until several years ago, when he committed suicide by hanging, without any known cause. He was, financially and otherwise, comfortably situated.

H. F. also had a paternal aunt that was at times insane. He was apparently well until nineteen years of age, when he became insane, and was for several months an inmate of the asylum at Utica. He afterward served his time as a machinist, and soon afterward got an appointment as locomotive engineer, a calling which he followed almost uninterruptedly until several years ago, when he was compelled to quit on account of impaired general health. Six months previous to his admission he had an apoplectic fit, followed by partial right hemiplegia, and considerable mental excitement, from which he recovered completely in the course of several weeks. He had several similar attacks previous to his admission, September 11th, 1879.

Sept. 13.—Had a fit last night; paralysis more marked. Mentally much depressed, speaks of committing suicide.

Sept. 19.—Since the 13th he has gradually become excitable and noisy. This A. M., is very violent, gesticulating with his sound arm and talking incessantly.

Sept. 24.—General health failing. Mentally depressed. Marked loss of memory.

Sept. 25.— Very noisy, shouting at the top of his voice.

Oct. 10.—Since September 25th has been violent most of the time. Sedatives, and, later, milk punches were administered without relieving the mental excitement or insomnia. I also injected $\frac{1}{8}$ grain of morphine hypodermically every four hours, which caused eight hours of sleep after the third injection.

Oct. 12.—Much better this A. M. For the first time since admitted is able to hold knife in paralyzed hand.

Oct. 14.—Walked several steps without support.

Oct. 20.—Marked mental improvement.

Nov. 1.—Able to walk about the ward without aid. Transferred to convalescent ward.

Nov. 17.—Had several fits last night. Hemiplegia returning.

Nov. 19.—Had five fits in one hour, yesterday.

Nov. 20.—Hemiplegia complete. Intellect not so much disturbed as when admitted.

Dec. 2.—Had no fits since November 20th, and has been improving ever since.

Dec. 5.—Paralysis disappearing ; is able to walk alone.

Dec. 20.—Had no fits the past month ; is able to be about all day. Is very talkative, and at times emotional.

Dec. 29.—Marked improvement. He is allowed the liberty of the grounds. From this date he improved rapidly, and had no more fits until he eloped January 15th, walking eight miles to Chicago, where he remained. On February 13th the fits returned with great frequency and severity, the hemiplegia manifesting itself after the first fit. He soon became unconscious, and died several minutes after a severe fit, February 15th.

This case, aside from its general interest, illustrates the positions of trust sometimes filled by persons of a highly insane neurusis. For many years he followed the calling of locomotive engineer, and when admitted he had in his possession letters of recommendation from railroad superintendents of some of the leading railroads in the United States. A previous attack of insanity, and a marked hereditary taint should hardly be regarded as qualities fitting a man to hold a position where the destinies of hundreds of lives are frequently placed in his sole care. How some sudden danger might have developed insanity is illustrated by the case of Von H., a German locomotive engineer, running out of Paris, France, who went insane during a railroad collision, the result of misinterpreted orders. He was for two and a half years an inmate of an asylum in Paris, when he was discharged as "recovered."*

The statement of M. Esquirol, that persons born before the development of insanity in the parents are less subject to mental disease than those who are born after the malady has displayed itself, I regard as due in some instances to the fact that the insanity in the parent may have originated after the birth of some of the children. In some cases, the children born previous to the development of insanity in the parents are completely free from hereditary taint. A patient treated here illustrates the point:

Case V.—J. B., aged seventeen years, admitted February 13, 1879, with the following history: He was always regarded as dull, and could not keep up with his classes while at school; was always quiet and inoffensive, preferring solitude to companionship. He had the exact features of his father, a sober, industrious man, with a good family history, whose sanity was never questioned until after he met with a severe accident, nine years previous to the birth of the patient, when he sustained a severe fracture of the skull as a result of a kick by a vicious horse he was grooming. He was carried to his home in an unconscious condition, in which he remained for several days. Upon regaining consciousness it was discovered that his mind was shattered to such an extent that he did not recognize his friends. He was sent to an asylum in Scotland, from which he was discharged greatly improved, at the end of seven years. Two years after his return home the subject of this history was born; shortly afterward his father grew worse, and was re-admitted to the asylum, where he died demented four years later. Three sisters and one brother to J. B., born previous to the injury to their father, which was regarded as the sole cause of his insanity, are living and well, presenting no perceptible indications of an in-

* The patient is at present an inmate of this asylum.

sane neurosis. If the insanity in the father was due entirely to the injury he sustained, the children born previous to that event are entirely free from an inherited neurosis. The form of insanity in J. B. was mania, at times very acute. He manifested homicidal tendencies, and imagined that he was followed by detectives seeking nis destruction. He commenced to improve shortly after his admission, and was discharged as "recovered" July 1st, 1879.

*Members of Insane Families not Themselves Insane.*—While certain members of a family predisposed to insanity become insane, others pass through life without insanity developing. Such persons are frequently spoken of by their associates as nervous, peculiar, or eccentric; they have unstable nervous systems, and poorly balanced intellects, escaping insanity in some instances by the absence of sufficient exciting causes. Innumerable examples of this class of persons are noticed among the blood relatives of the insane while visiting the asylum. No one who has come in contact with these people, especially in asylums, where they are unusually excited, but has had occasion to overlook in them undue excitement, rude manners, and uncalled for criticisms, by remembering that they too were the victims of an inherited predisposition to mental disease, awaiting, perhaps, some trifling cause to develop active insanity. Some of these people are a source of great annoyance to asylum authorities, rushing into print and telling unheard of stories of cruelty and neglect, which are generally proven entirely false if thoroughly investigated. An unusually troublesome relative of this class has frequently complained of the management of every asylum her relative was ever an inmate of, going so far as to interview personally nearly every member of the board of commissioners, writing articles to the public press, all because her son did not display the amount of adipose she had expected he would.

*Exciting Causes.*—The exciting causes in persons hereditarily predisposed to mental disease are very variable. In some instances the most trivial causes, such as would not affect a healthy mental organization are sufficient to destroy the equilibrium of these poorly balanced minds. To illustrate what slight causes are sometimes sufficient to develop insanity in such persons, I

will briefly report two cases that came under my observation in this hospital:

Case VI.—J. W., an individual of this class who had a sister, uncle and several more distant blood relatives insane, had a son meet with a railroad accident, necessitating the amputation of one of his arms. He refused to be consoled, quit his work, and has never worked any since. That the father of a large family depending upon his daily labor for support should refuse to be comforted and lose his reason because of an accident (not fatal) to one of his sons is strong evidence of his unstable nervous organization. The increased responsibilities would have stimulated a healthy mind to greater exertions to provide for his family, and alleviate the sufferings of the son.

Case VII.—P. B., aged eighteen years, whose father was insane for a short period, and an inmate of an asylum forty years ago, whose oldest brother committed suicide by dividing the radial artery and bleeding to death, whose maternal grandmother was insane for thirty years previous to her death, and whose mother, for many years previous to his birth, was at times insane, was admitted to the asylum April 15, 1880. It will be seen that several ancestors on paternal and maternal sides were insane, the entire history pointing to a strongly inherited neurosis. Eleven months previous to his admission here he arrived in America, and almost immediately afterward found employment, but entertaining the most extravagant ideas of the ease with which fortunes were amassed in America, he became greatly depressed on finding that he had to work hard to provide the necessities of life. He conceived the idea that God had forsaken him, and that having to contend against the displeasure of his Maker, he had better sacrifice his life to appease His wrath. With this end in view he tried every means at his command to put an end to his existence, abstaining from all food and constantly looking for an opportunity to commit suicide. The predisposition to mental disease must indeed be strong in a young man with no one but himself to provide for, and without meeting with any of the innumerable hardships through which persons with well-balanced minds frequently pass without the slightest danger to their mental equilibrium, allows the ordinary disappointments which every immigrant has to overcome, so to prey upon his mind that he believes himself the most unfortunate of beings, whom the very Deity has forsaken. When slight causes, such as would make no unfavorable impression upon a healthy mind are the exciting causes of insanity, it is always well to look carefully for an inherited predisposition to insanity.

In a number of cases in which especial attention was paid to exciting causes, it was noticed that the greater the inherited predisposition, the milder the causes sufficient to develop insanity.

The following table will show the supposed exciting causes in fifty male patients admitted with a history of hereditary predisposition :

TABLE II.

| | | | |
|---|---|---|---|
| Disappointed affections | 1 | Masturbation | 6 |
| Domestic troubles | 5 | Overwork | 1 |
| Epilepsy | 4 | Poverty | 3 |
| Excesses | 2 | Religious excitement | 3 |
| Financial | 10 | Traumatic | 1 |
| Grief | 1 | | |
| Intemperance | 13 | Total | 50 |

In thirty-four cases, or a trifle over seventy-five per cent. of the total number, intemperance, financial troubles, masturbation and domestic troubles were regarded as exciting causes. Intemperance was an exciting cause in thirteen cases, or over twenty-five per cent. It would be interesting to know in what percentage, if any, the taste for alcoholic stimulants was hereditary, but unfortunately the histories do not embrace this point. Financial troubles were exciting causes in ten instances. Reverses in fortune are especially hard to bear by persons of delicate mental organizations, hence their frequency as exciting causes. Instead of the reaction which usually follows in healthy minds, in the naturally weak textural changes take place, which interfere with or prevent reaction taking place. In this manner persons of this class have been known to become permanently demented immediately after sudden reverses in fortune.

Masturbation was regarded as an exciting cause in six cases. I have no doubt that the inherited nervous temperament in some of these led to the practice of the vice ; add to this an inherited weakness of will power, and we can understand why when once learned, the practice is rarely discontinued before the mental faculties become greatly impaired or totally destroyed.

In the five cases in which domestic troubles were regarded as the exciting causes, in two instances the family troubles were directly traceable to the nervous temperament and abnormal conduct of the party with an inherited neurosis.

A case illustrating how an insane temperament may cause domestic troubles, came to my notice in a patient who was recently discharged as "recovered," in whom the exciting cause was regarded as domestic troubles, because he and his wife had led very unhappy and quarrelsome lives for several years previous to his admission to the asylum, and long before he was suspected of being of unsound mind. Just previous to his discharge, and when fully restored to reason he informed me one day that he would tell me the cause of his unhappy domestic relations. He said: "I am naturally of a jealous disposition, and when I married a wife ten years my junior I was very jealous of her. On returning from work one evening, several years ago, I found her sitting in the parlor entertaining a gentleman friend of the family's who had taken the trouble to look us up and make a call. The moment I saw him with my wife the idea entered my mind that he had called upon her in my absence for improper purposes."

From that moment he suspected his wife of being unfaithful, and watched her every action. His wife, noticing his changed manner toward her, unconscious of any wrong on her part, and not aware of his weak mental condition, resented his slights and insinuations, and hence their unhappy domestic relations. The case entered as traumatic is that of a patient who became insane during an attack of erysipelas of the face and head following an injury.

*General Health.*—The general health is almost invariably somewhat impaired in persons hereditarily predisposed to insanity, especially after the development of the malady; so frequently is this the case that in seventeen of the forty-one cases recorded in Table I, the general health was greatly impaired. The following physical peculiarities were also noted:

In case 1, general anæmia, right hemiplegia. In case 4, general anæmia, neurasthenia. In case 5, emaciation, inanition, vaso-motor paralysis. In case 6, general anæmia. In case 7, partial muscular paralysis, both extremities. In case 9, irregular dilatation of pupils, locomotor ataxia. In case 11, irregular dilatation of pupils, frontal headache. In case 13, headache, not localized; tongue tremulous. In case 14, tongue enlarged, indentation of teeth along the sides. In case 15, anæmia, neurasthenia. In case 16, constant frontal headache, valvular heart lesions. In case 18, intermittent headache. In case 18, neurasthenia; indentation of teeth sides of tongue.

Cases 11, 16, and 18 complained of headache. This percentage is considerably larger than among the insane generally. It is remarkable how seldom the inmates of asylums complain of headache. At the present writing there are but two male patients in the entire asylum who have sufficient pain in the head to cause them to apply for relief, and during the past three months I have had more calls from patients themselves to prescribe for colds than headaches. And even persons in whom headache is a prominent symptom previous to the development of insanity are not infrequently entirely free from this symptom during an attack.

Case VII.—O. B., printer, lost several days each month on account of severe headaches, which obliged him to take to bed. He has not complained of this symptom since admitted to the asylum several months ago, and just previous to his discharge as "recovered" he informed me that he had had no "pain in the head" since admitted, and that the only head symptom he experienced was a feeling of fullness, as if the brain was larger and heavier than formerly. This sensation was no doubt due to cerebral congestion. General, and especially cerebral anæmia is a frequent symptom. In melancholy patients the anæmia frequently, is a result of an insufficient quantity of nourishment taken for weeks, and even months previous to complete abstinence, imagining that the food was poisoned, or mixed with disgusting matter, such as human flesh or even feces. Six of the patients in Table I were neurasthenic previous to being regarded as insane. This is quite a common affection among persons who have inherited an unstable nervous system. That the entire nervous system is predisposed to disease is proven by the prevalency of hysteria, epilepsy, chorea, and other nervous affections among the blood relatives of the hereditary insane. Constipation is frequently met with in cases of melancholia and acute mania, and a marked mental improvement sometimes follows the administration of a brisk cathartic. Some of the remaining most constant symptoms are vasor-motor paralysis and enlarged, coated or tremulous tongue.

*Peculiarities of Development.* The arrest of cranial and sometimes general development among imbeciles, idiots, and especially "cretins" are so marked that there is no possibility of mistaking them. A large percentage of the feeble-minded children in the asylums for the feeble-minded present physical defects in the cranial and general development, but the percentage of deformed feeble-minded children is much greater outside these institutions, which refuse to receive epileptic or greatly deformed idiots, as the institutions are intended for that class of feeble-minded children only who, with proper instruction and training, are capable of becoming self-supporting under proper supervision ; this, of course, excludes all the lower grades of idiots, who are most frequently deformed. The physical defects among idiots of the lowest grade are frequently so marked as to suggest at a glance, even to the minds of people outside of the profession, imperfect cerebral development. If these patients, who are not infrequently the offspring of insane parents, carry in their very appearance a diagnosis of their disease, it would seem most probable that the inheritors of an unstable nervous system, predisposed to mental disease, would present some physical peculiarities characteristic of an inherited neurosis.

To determine what physical peculiarities, if any, existed among this class of people, I prepared the two following tables, which show the cranial measurements, height, weight, color of eyes, color of hair, and asymmetrical development, of forty insane patients, in twenty of whom the disease was regarded as having originated *per se*, with the following results :

### TABLE III.

INSANITY HEREDITARY.

| No. | "1." | "2." | "3." | "4." | "5." | "6." | "7." | "8." | "9." | "10." |
|---|---|---|---|---|---|---|---|---|---|---|
| 1 | 22¼ | 12 | 11 | 11 | 13¼ | 5.11 | 150 | Black. | Brown. | |
| 2 | 22¼ | 12½ | 11½ | 11 | 13½ | 5.8 | 145 | Brown. | Brown. | Fullness right side of head. |
| 3 | 21½ | 12¼ | 11 | 10½ | 13 | 5.7 | 155 | Brown. | Blue. | |
| 4 | 21¾ | 12 | 11¼ | 10½ | 14 | 5.7 | 155 | Brown. | Brown. | Left frontal and occipital contr'c'n. |
| 5 | 22 | 13 | 11½ | 11 | 13¼ | 5.9 | 130 | Black. | Brown. | |
| 6 | 22½ | 13 | 11½ | 11 | 14 | 6.1 | 165 | Brown. | Blue. | |
| 7 | 22½ | 12 | 11½ | 10½ | 13 | 5.5½ | 135 | Brown. | Brown. | Rt. frontal contraction. |
| 8 | 21¾ | 12 | 11 | 12¾ | 12¼ | 5.10 | 160 | Light. | Blue. | Fullness right side of head. |
| 9 | 23 | 13 | 12 | 12 | 13¾ | 5.7½ | 145 | Light. | Blue. | |
| 10 | 22¾ | 13 | 12 | 10½ | 13½ | 5.9½ | 158 | Brown. | Brown. | |
| 11 | 22 | 12 | 11½ | 10 | 13½ | 5.8 | 130 | Brown. | Blue. | Left frontal fullness. |
| 12 | 21½ | 13 | 11¼ | 9½ | 13½ | 5.4¾ | 118 | Brown. | Brown. | Left frontal fullness. |
| 13 | 22 | 12 | 11 | 12 | 13½ | 5.9 | 135 | Gray. | Blue. | |
| 14 | 21½ | 12½ | 11½ | 10½ | 13 | 5.8 | 135 | Brown. | Blue. | |
| 15 | 22½ | 14¾ | 12 | 10 | 14½ | 5.2 | 123 | Light. | Blue. | Parietal bulging. |
| 16 | 22 | 12¾ | 11 | 12½ | 14 | 5.4 | 130 | Gray. | Blue. | |
| 17 | 22 | 11½ | 11 | 11½ | 13 | 5.6½ | 135 | Gray. | Blue. | |
| 18 | 22¼ | 12 | 11½ | 11 | 14 | 5.9 | 170 | Brown. | Brown. | |
| 19 | 22½ | 12½ | 12 | 10½ | 14 | 5.9 | 145 | Black. | Brown. | Contr'c'n right part parietal region. |
| 20 | 23 | 13 | 12 | 11 | 14¼ | 5.8 | 175 | Brown. | Blue. | Left parietal contraction. |
| | 22⅛ | 12½ | 11½ | 11 | 13½ | 5.7½ | 144 7-10 | | | —Average. |

### TABLE IV.

INSANITY NOT HEREDITARY.

| No. | "1." | "2." | "3." | "4." | "5." | "6." | "7." | "8." | "9." | "10." |
|---|---|---|---|---|---|---|---|---|---|---|
| 1 | 21½ | 12½ | 12 | 10 | 13 | 5.2½ | 120 | Brown | Blue. | |
| 2 | 22 | 12¼ | 12¾ | 10½ | 13 | 5.8 | 145 | Brown. | Blue. | |
| 3 | 22 | 12½ | 11¼ | 10½ | 12¼ | 5.6½ | 135 | Black. | Brown. | |
| 4 | 23¼ | 12½ | 11¼ | 11¾ | 12½ | 5.5¾ | 170 | White. | Blue. | |
| 5 | 22 | 12 | 11 | 10¼ | 13¼ | 5.11 | 160 | Brown. | Blue. | |
| 6 | 22½ | 12½ | 11¾ | 10½ | 12½ | 5.8 | 140 | Brown. | Brown. | |
| 7 | 23½ | 13 | 12 | 11 | 13 | 5.6½ | 155 | Brown. | Blue. | |
| 8 | 21 | 12 | 11 | 10 | 13 | 5.4½ | 130 | Brown. | Blue. | |
| 9 | 21½ | 12 | 11 | 10¼ | 12¼ | 5.5 | 135 | Brown. | Brown. | |
| 10 | 23¼ | 13 | 11½ | 12¼ | 14½ | 6. | 170 | Light. | Blue. | |
| 11 | 23¾ | 13⅛ | 12¼ | 11 | 13 | 5.6½ | 180 | Brown. | Blue. | |
| 12 | 22¾ | 13 | 11 | 11¾ | 14 | 5.7¼ | 145 | Sandy. | Blue. | |
| 13 | 22⅛ | 12 | 11¾ | 11 | 13 | 5.9 | 140 | Brown. | Blue. | Contracted right eye and forehead. |
| 14 | 21⅜ | 13 | 11½ | 10¾ | 14 | 5.9 | 140 | Grey. | Blue. | |
| 15 | 23½ | 13¼ | 13 | 10½ | 14 | 5.9 | 155 | Brown. | Blue. | Parietal region contracted. |
| 16 | 21¾ | 14 | 11¼ | 10¼ | 14¼ | 5.9 | 140 | Brown. | Blue. | Parietal region bulging. |
| 17 | 22¼ | 12½ | 11½ | 10 | 14¼ | 5.5 | 135 | Light. | Blue. | |
| 18 | 21¾ | 12½ | 11⅛ | 10½ | 13¾ | 5.5 | 130 | Brown. | Blue. | Fullness entire left side. |
| 19 | 22¼ | 11½ | 11 | 11 | 13½ | 5.6 | 145 | Brown. | Blue. | |
| 20 | 22 | 12¼ | 11½ | 11 | 14 | 5.8 | 150 | Brown. | Blue. | |
| | 22¼ | 12½ | 11½ | 10¾ | 13⅜ | 5.7 | 146 | | | —Average. |

### EXPLANATION OF TABLES III AND IV.

"1." Circumference of head on a line with nasal eminence and ext. occip. protuberance.

"2." From ear to ear over parietal region.

"3," From ear to ear over nasal eminence.

"4." From ear to ear over ext. occip. protuberance.

"5." From nasal spine to ext. occip. protuberance.

"6." Height.

"7." Weight.
"8." Color of hair.
"9" Color of eyes.
"10." Asymmetrical development.

By comparing the tables it will be seen that the average cranial measurements are very similar, and do not show any difference in the general size of the crania. The "height" and "weight" were remarkably similar, there being an average difference of less than two pounds in the weight, and but four-fifths of an inch in the height, showing that there is no general arrest of development characteristic of an inherited insane neurosis, and that while the nerve tissue may be ever so predisposed to disease, there generally is an average cranial development.

While the tables do not show any general arrest of development, they do show a marked difference in the asymmetrical development. Thus in Table IV the cranial development was symmetrical in all but cases 13, 15, 16 and 18, while in Table III an asymmetrical development was noticed in nine cases, nearly fifty per cent. of the total number examined.

The unequal developments in Table IV were as follows:

Case 13.—Contracted right eye and right side of forehead.

Case 15.—(An epileptic patient) parietal regions contracted; more marked on left side.

Case 16.—Parietal regions bulging; more marked on right side.

Case 18.—Entire left side of head enlarged.

In Table III they were:

Case 2.—Right side of head enlarged, especially noticeable in frontal region.

Case 4.—Left frontal and occipital contraction.

Case 7.—Right frontal contraction.

Case 8.—Fullness entire right side of head.

Case 11.—Left frontal fullness.

Case 12.—Left frontal fullness.

Case 19.—Contraction right part, posterior parietal region.

Case 20.—Left parietal contraction.

Aside from the asymmetrical cranial development there is frequently a difference in the development of the two sides of the face, especially noticeable in the wrinkles on the forehead, and the lines extending from the alæ of the nose to the angle of the mouth, so that in some instances the patient presents an appearance somewhat similar to that seen in hemiplegic persons where the muscles of one side of the face are slightly involved. While the above peculiarities of asymmetrical development are most frequently met with among the insane in whom the malady is hereditary, they are all met with in persons of sound mind without any inherited or acquired predisposition to insanity. A want of symmetry in the cranial development cannot therefore be regarded as an invariable evidence of an insane neurosis or liability to mental disease, and yet its greater frequency among the hereditary insane should not be overlooked as an element in forming an opinion as to the probability of insanity developing as well as suggest early treatment to avert an outbreak of insanity.

### TABLE V.

INSANITY HEREDITARY.

| No. | Recovered. | Improved. | Unimproved. | Died. | Eloped. | Number of months in Asylum. | Re-admitted. | Sent to State Hospital. | Here. |
|---|---|---|---|---|---|---|---|---|---|
| 1 | ...... | ...... | ...... | ...... | 1 | 4 | ...... | ...... | ...... |
| 2 | 1 | ...... | ...... | ...... | ...... | 8 | ...... | ...... | ...... |
| 3 | 1 | ...... | ...... | ...... | ...... | 4 | ...... | ...... | ...... |
| 4 | ...... | ...... | ...... | ...... | ...... | 10 | ...... | 1 | ...... |
| 5 | ...... | 1 | ...... | ...... | ...... | 5 | ...... | ...... | ...... |
| 6 | ...... | ...... | ...... | 1 | ...... | 1 | ...... | ...... | ...... |
| 7 | ...... | 1 | ...... | ...... | ...... | 2 | 1 | ...... | ...... |
| 8 | ...... | 1 | ...... | ...... | ...... | 2 | ...... | ...... | ...... |
| 9 | ...... | ...... | ...... | 1 | ...... | 4 | ...... | ...... | ...... |
| 10 | ...... | 1 | ...... | ...... | ...... | 3 | 1 | ...... | ...... |
| 11 | ...... | 1 | ...... | ...... | ...... | 2 | 1 | ...... | ...... |
| 12 | ...... | ...... | ...... | 1 | ...... | 4 | ...... | ...... | ...... |
| 13 | ...... | 1 | ...... | ...... | ...... | 1 | 1 | ...... | ...... |
| 14 | ...... | ...... | 1 | ...... | ...... | 4 | ...... | ...... | ...... |
| 15 | ...... | ...... | ...... | ...... | ...... | ...... | ...... | ...... | 1 |
| 16 | ...... | ...... | ...... | ...... | ...... | 3 | ...... | 1 | ...... |
| 17 | 1 | ...... | ...... | ...... | ...... | 1 | ...... | ...... | ...... |
| 18 | 1 | ...... | ...... | ...... | ...... | 3 | ...... | ...... | ...... |
| 19 | ...... | 1 | ...... | ...... | ...... | 4 | ...... | ...... | ...... |
| 20 | ...... | 1 | ...... | ...... | ...... | 4 | ...... | ...... | ...... |
| 21 | ...... | ...... | ...... | ...... | ...... | 8 | ...... | 1 | ...... |
| 22 | ...... | 1 | ...... | ...... | ...... | 1 | 1 | ...... | ...... |
| 23 | ...... | ...... | 1 | ...... | ...... | 3 | ...... | ...... | ...... |
| 24 | ...... | ...... | ...... | ...... | ...... | 7 | ...... | 1 | ...... |
| 25 | ...... | ...... | ...... | 1 | ...... | 5 | ...... | ...... | ...... |
| 26 | ...... | ...... | ...... | 1 | ...... | 7 | ...... | ...... | ...... |
| 27 | ...... | ...... | ...... | ...... | 1 | 8 | ...... | ...... | ...... |
| 28 | 1 | ...... | ...... | ...... | ...... | 3 | 1 | ...... | 1 |
| 29 | ...... | ...... | 1 | ...... | ...... | 6 | 1 | ...... | 1 |
| 30 | 1 | ...... | ...... | ...... | ...... | 3 | ...... | ...... | ...... |
| 31 | ...... | ...... | ...... | 1 | ...... | 4 | ...... | ...... | ...... |
| 32 | 1 | ...... | ...... | ...... | ...... | 4 | ...... | ...... | ...... |
| 33 | ...... | 1 | ...... | ...... | ...... | 6 | 1 | ...... | ...... |
| 34 | 1 | ...... | ...... | ...... | ...... | 5 | ...... | ...... | ...... |
| 35 | ...... | 1 | ...... | ...... | ...... | 1 | 1 | ...... | ...... |
| 36 | ...... | ...... | ...... | 1 | ...... | 3 | ...... | ...... | ...... |
| 37 | ...... | ...... | 1 | ...... | ...... | 2 | ...... | ...... | ...... |
| 38 | ...... | 1 | ...... | ...... | ...... | 24 | 1 | ...... | ...... |
| 39 | ...... | 1 | ...... | ...... | ...... | 24 | 1 | ...... | ...... |
| 40 | ...... | 1 | ...... | ...... | ...... | 4 | 1 | ...... | ...... |
| Total | 8 | 14 | 4 | 7 | 2 | | 12 | 4 | 5 |

*Prognosis.* The prognosis as to a first recovery would seem to be more favorable in persons hereditarily predisposed, as will be seen by comparing Tables V. and VI. comprising forty patients of either class.

### TABLE VI.

INSANITY NOT HEREDITARY.

| No. | Recovered. | Improved. | Unimproved. | Died. | Eloped. | Number of months in Asylum. | Re-admitted. | Sent to State Hospital. | Here. |
|---|---|---|---|---|---|---|---|---|---|
| 1 | | | | | | | | | 1 |
| 2 | | | | 1 | | 1 | | | |
| 3 | | | | | 1 | 10 | 1 | | 1 |
| 4 | 1 | | | | | 1 | | | |
| 5 | | | | | | | | | 1 |
| 6 | | | 1 | | | 4 | | | |
| 7 | | | | 1 | | 1 | | | |
| 8 | 1 | | | | | 1 | | | |
| 9 | | | | 1 | | 1 | | | |
| 10 | | 1 | | | | 12 | | | |
| 11 | 1 | | | | | 3 | | | |
| 12 | | 1 | | | | 4 | 1 | | |
| 13 | | | | 1 | | 1 | | | |
| 14 | | 1 | | | | 3 | | | |
| 15 | | | 1 | | | 9 | 1 | | 1 |
| 16 | | | | | | | | | 1 |
| 17 | | | | | | 2 | | 1 | |
| 18 | | | | 1 | | 20 | | | |
| 19 | | 1 | | | | 3 | | | |
| 20 | | | | | 1 | 3 | | | |
| 21 | | 1 | | | | 12 | 1 | | |
| 22 | 1 | | | | | 2 | | | |
| 23 | | | | | | 1 | | 1 | |
| 24 | 1 | | | | | 3 | | | |
| 25 | | 1 | | | | 1 | | | |
| 26 | | | | | | 8 | | 1 | |
| 27 | 1 | | | | | 3 | | | |
| 28 | | | | | | | | | 1 |
| 29 | | | | | | | | | 1 |
| 30 | | | | | | 5 | | 1 | |
| 31 | | | | 1 | | 1 | | | |
| 32 | | | | 1 | | 1 | | | |
| 33 | | | | | | | | | 1 |
| 34 | | | | | | | | | 1 |
| 35 | | 1 | | | | 10 | 1 | | 1 |
| 36 | | | | 1 | | 2 | | | |
| 37 | | 1 | | | | 4 | | | |
| 38 | | 1 | | | | 18 | | | |
| 39 | | 1 | | | | 10 | 1 | | 1 |
| 40 | | 1 | | | | 2 | | | |
| Total | 6 | 11 | 2 | 8 | 2 | | 6 | 4 | 11 |

INSANITY HEREDITARY.

Average number of months in asylum of those discharged, recovered, 3⅞.

Average number of months in asylum of those discharged improved, 5⅞.

Average number of months in asylum of those discharged unimproved, 3 4-5.

Average number of months in asylum of those who died, 4.

Average number of months of those sent to State Hospital, 6.2-5.

INSANITY NOT HEREDITARY.

Average number of months in asylum of those discharged recovered, 2.

Average number of months in asylum of those discharged improved, 11 2-7.

Average number of months in asylum of those discharged unimproved, 6½.

Average number of months in asylum of those who died, 3½.

Average number of months of those sent to State Hospital, 5⅜.

RECAPITULATION OF TABLE V.

Discharged recovered, 8; improved, 14; unimproved, 4; died, 7; eloped, 2; re-admitted, 12; sent to State Hospital, 4; remaining, 5.

RECAPITULATION OF TABLE VI.

Discharged recovered, 6; improved, 11; unimproved, 2; died, 8; eloped, 2; re-admitted, 6; sent to State Hospital, 4; remaining, 11.

By comparing Tables V and VI, it will be seen that twenty-two of the former, and seventeen of the latter were discharged as recovered and improved, tending to show that the percentage of first discharges are in favor of the former class. While the percentage of discharges was greater in the former, it will be noticed in the same tables that the improvement was less permanent, as twelve of the former and but six of the latter were re-admitted to the hospital. The principal reason of this fact I regard as the difference in the exciting causes sufficient to produce insanity in the two classes, as well as the impossibility of removing the inherited predisposition to mental disease in the first class. And even when a cure is effected in the former, that is, when the brain is restored to its normal action, some slight excitement may at any time disturb its equilibrium, while in the patients not hereditarily predisposed, if the brain can be restored to its normal condition, it is able to resist an exciting cause, that would be sufficient to again disturb a naturally defective organ. The number of deaths were nearly the same in both classes. The causes of death call for no special explanation, excepting case 13 of Table VI, where suicide by hanging caused death by strangulation. The average time in asylums of those discharged "recovered" was considerably longer in the former class. The only explanation of this fact that I can advance is the relative recuperative powers of a naturally healthy and a naturally weak organ just as a pair of naturally weak lungs are longer in completely recovering from an attack of pneumonia or bronchitis, than vigorous, healthy organs. According to the tables, a larger number of the second class become permanent charges of insane asylums, as five of the former and eleven of the latter at present remain in the asylum.

The conclusions drawn from the tables are that patients with an inherited neurosis have a greater chance of a first discharge than others, but that they are more liable to relapse, owing to an inherited unstable nervous system.

*Treatment.*—The preventive treatment is generally in the hands of the general practitioner, hence a knowledge of the manifestation of the disease and the treatment adopted after the case passes out of their hands into that of the specialist in some hospital for the insane cannot fail to be of interest. Preventive treatment should commence with infancy and extend through life, and should aim to meet the following indications:

First. Improve the general health.

Second. By judicious training strengthen the mental faculties, the better to enable them to resist disease.

Third. Guard against excesses and abuses, especially of the sexual organs.

Fourth. Place them in positions where they can avoid all sources of excitement and excessive mental work.

To improve the general health, which (as has already been stated), is generally impaired, is of the greatest importance, as just in proportion to the improved health is the nervous system able to resist disease. Of all the means at our command to improve the general health of a nervous child nothing can compare with plenty of pure air, out-door exercise, and plain, unstimulating diet, as may be seen in the improvement in appearance, appetite, vigor and spirits of a nervous city child on returning from a summer vacation in the country; and even later in life, when from close confinement and overwork the general health becomes impaired, what can compare with a country vacation for the class that cannot afford the luxury of foreign travel?

The mind may be strengthened, and better enabled to resist disease by a careful training during the period of development, while the character is being formed. The greatest care should be exercised in selecting associates, teachers and schools for the persons predisposed to mental disease. The ordinary boarding schools (especially for females), would seem to be about the most objectionable that could possibly be selected. In proof of this assertion, notice the anæmic, and at times neurasthenic condition of the average female graduate. A school should be selected in a healthy locality, where due regard is paid to physical exercise, with positive instructions not to allow over-study. For the children of the poorer classes who attend school at intervals only for a few years before they are called upon to assist in the support of the family, the public schools afford the only means of acquiring even a rudimentary education. Previous to the ages when children are sent to school, in fact from the earliest manifestation of the emotions and passions a careful watch should be exercised, to avoid all causes of excessive emotional outbreaks, and firmly checking them when displayed without cause. Even in very small children bursts of passion can be controlled by firmness on the part of parents, which, if yielded to might lead to a life of misery and mental disease. "The habit of yielding to a natural infirmity of temper often leads into paroxysms of ungovernable rage, which, in their turn, pass into maniacal excitement."*

The abuse of the sexual organs I believe to be more frequently indulged in, and more serious to the mental health of nervous children than others, hence great care should be exercised in excluding every cause tending to stimulate the sexual passions; obscene literature, improper associates, stimulants and sedentary habits should be carefully guarded against. A plain statement on the part of the parents or physician as to the true functions of the organs of reproduction and the dangers to health and happiness following their abuse should be enforced. Every cause calculated to unduly stimulate or to direct the attention of an excitable youth to the genitalia should be guarded against.

To place persons predisposed to insanity in positions where they can avoid excessive mental work and sources of dangerous excitement, great care should be exercised in the selection of a trade or profession. No rules governing every case can be laid

* Carpenter's Physiology.

down; as no two persons are exactly alike, so every case must be made a special study of, and every possible influence bearing on the case must be taken into consideration in making a selection. It is safe in every case, however, to avoid callings requiring severe continued mental work, or one subject to great uncertainties and sudden reverses. In fact, the quieter such persons pass through life, and the more they avoid all sources of excitement, the greater are their chances of passing through life without any manifestation of insanity.

The curative treatment is usually administered in hospitals for the insane, as but a very small percentage of cases amenable to treatment could possibly be treated at home, owing to the acute nature of the symptoms. The treatment is necessarily very different even in similar forms of insanity, owing to the difference in the general health, age, etc. The same general principles which govern the treatment of diseased conditions in other organs should be carefully observed. Congestion is to be relieved. In anæmia, food and medicine calculated to improve the quality of the blood are to be prescribed. One of the chief duties of the general practitioner when called upon to examine an insane person is to decide whether home treatment, or removal to an asylum is to be recommended. As a rule, a removal of the patient from home and friends is to be advised, as they not infrequently have some delusions regarding some member of the family or dear friend, whose constant presence and efforts to alleviate the sufferings of the patient are misconstrued into evidences of their insane imaginations. I have seen a number of patients admitted to asylums hopelessly insane, who, by a timely removal to some retreat would undoubtedly have recovered. The mistaken kindness of keeping insane persons at home has protracted or prevented many a cure. Any physician who has been called upon to treat many cases of acute melancholia will bear me out in this statement, when he recalls the difficulties he encountered in attempting to administer food or medicine to these patients, surrounded by sympathizing friends, and should a patient refuse food entirely, and the use of a stomach tube become necessary, it would be almost impossible in some families to get the necessary assistant to perform even this simple operation. The danger of homocidal or suicidal tendencies developing must be taken into consideration. From what I have seen of insane persons outside of asylums, especially among the poor, I have become satisfied that the chances of recovery are far greater in asylums than at home. It must also be remembered that the blood relatives of a person suffering from an inherited disease are themselves tainted with the same affection, hence the dangers to them of home treatment must not be overlooked in deciding upon the course to be pursued. The annoyance and danger to society of a raving maniac, making night hideous with his cries, and day dangerous by his effort to carry out some homicidal delusion is also a very important factor to take into consideration, and it not infrequently transpires that society, recognizing these dangers, enter complaint and cause an investigation into the sanity of persons, against the expressed wishes of their friends. There is one important factor in the treatment of the chronic insane with considerable physical strength that is not sufficiently called into requisition, viz., active out-door exercise. I have noticed that patients who are daily employed in out-door work almost invariably improve while thus employed. I called attention to this fact in an article on "Masturbation Among the Insane," in the October (1879) number of the *Journal of Nervous and Mental Disease*, as follows: "Exercise carried to fatigue I regard as an important element in the treatment; daily out-door employment should be insisted on if practicable. Several patients of this class derived marked benefit last summer while digging a long ditch; the work was laborious, and while it prevented masturbation during the day, it produced such fatigue that they were glad to rest at night."

Since then I have had abundant opportunities to notice the good results following out-door employment in all forms of chronic insanity when the physical condition would permit its employment. A year ago about thirty patients were employed nearly four months digging trenches for a sewer over four miles long. At present, twenty-five patients are daily employed in excavating on the site for the new Cook County Infirmary. Patients with chronic mania of many years duration, in whom the prognosis as to recovery was regarded as very unfavorable, have been discharged as recovered after several months of active out-door employment. One of these cases was quite remarkable, and is briefly reported.

Case VIII.—J. K., aged twenty-eight years, admitted February 18, 1873, entered in the register as "mania," was for years the most unmanageable patient in the entire institution, and dreaded by the attendants on account of his violence and great strength. After a few years he settled down into chronic mania of rather a violent type, in which condition he remained without any apparent change until the summer of 1879, when he worked steadily for four months digging a ditch. While thus employed he commenced to improve, the improvement continuing the following winter, and he was discharged recovered April 24, 1880. When I last heard from the patient, about six months ago, he was working every day, enjoying excellent general health. A number of equally interesting cases could be cited if space would permit.

Insane persons on arriving at the asylums as inmates are usually carefully examined, and everything with which they could injure themselves or others is removed. They then take a bath, after which they are classified, or in some institutions sent to the ward for new patients. In this institution the patients are classified into eight grades (some hospitals have more), ranging from the convalescent ward to the ward for chronic dements. While it is true that "asylum life as at present constituted is unnatural, narrow, barren and mechanical," yet the asylums of the country afford places of shelter and safety to many thousands of diseased and troubled minds which it would be impossible to care for at home, and let us hope that the day is not far distant when the system for caring for the insane shall be so improved as to realize the highest ideal possible of attainment.*

* See Dr. Dewey's article on "The Present and Prospective Management of the Insane."—*Journal of Nervous and Mental Disease*, 1878.

